# A 61-year-old woman with jejunal lymphatic malformation visualized on computed tomography: a case report

**DOI:** 10.1186/s13256-021-02872-9

**Published:** 2021-05-27

**Authors:** Mark Rupasinghe, Roozbeh Houshyar, Chantal Chahine, Thanh-Lan Bui, Justin Glavis-Bloom, Caleb Cheng, Jill Tseng

**Affiliations:** 1 Department of Radiological Sciences, University of California Irvine, 101 The City Drive South, Route 140, Orange, CA 92868 USA; 2 Department of Pathology & Laboratory Medicine, University of California Irvine School of Medicine, Irvine, CA 92697 USA; 3 Department of Obstetrics and Gynecology, University of California Irvine, 333 City Boulevard West, Suite 1400, Orange, CA 92868 USA

**Keywords:** Lymphatic malformation, Cytoreduction surgical procedures, Diagnostic imaging

## Abstract

**Background:**

Jejunal lymphatic malformations are congenital lesions that are seldom diagnosed in adults and rarely seen on imaging.

**Case presentation:**

A 61-year-old Caucasian woman was initially diagnosed and treated for mucinous ovarian carcinoma. After an exploratory laparotomy with left salpingo-oophorectomy, a computed tomography scan of the abdomen and pelvis demonstrated suspicious fluid-containing lesions involving a segment of jejunum and adjacent mesentery. Resection of the lesion during subsequent debulking surgery revealed that the lesion seen on imaging was a jejunal lymphatic malformation and not a cancerous implant.

**Conclusions:**

Abdominal lymphatic malformations are difficult to diagnose solely on imaging but should remain on the differential in adult cancer patients with persistent cystic abdominal lesions despite chemotherapy and must be differentiated from metastatic implants.

## Background

Lymphatic malformations, previously known as “lymphangiomas,” are developmental anomalies of lymphatic ducts that are more commonly found in the head, neck, or axilla. Abdominal lymphatic malformations are exceptionally rare and account for less than 1% of all lymphatic malformations [1]. They can present with symptoms ranging from mild abdominal pain to an acute abdomen. Although lymphatic malformations directly associated with the bowel are rarely, if ever, imaged, computed tomography (CT) occasionally reveals cystic structures on the mesentery that compress adjacent bowel [2]. A definitive diagnosis is typically achieved after surgical excision and histologic examination demonstrate dilated lymphatic channels notable for continuous, flat endothelia [3]. In this case report, we present a 61-year-old woman whose exceptionally rare jejunal lymphatic malformation was seen on CT but was initially believed to be a peritoneal implant from her ovarian carcinoma.

## Case presentation

**Fig. 1.**
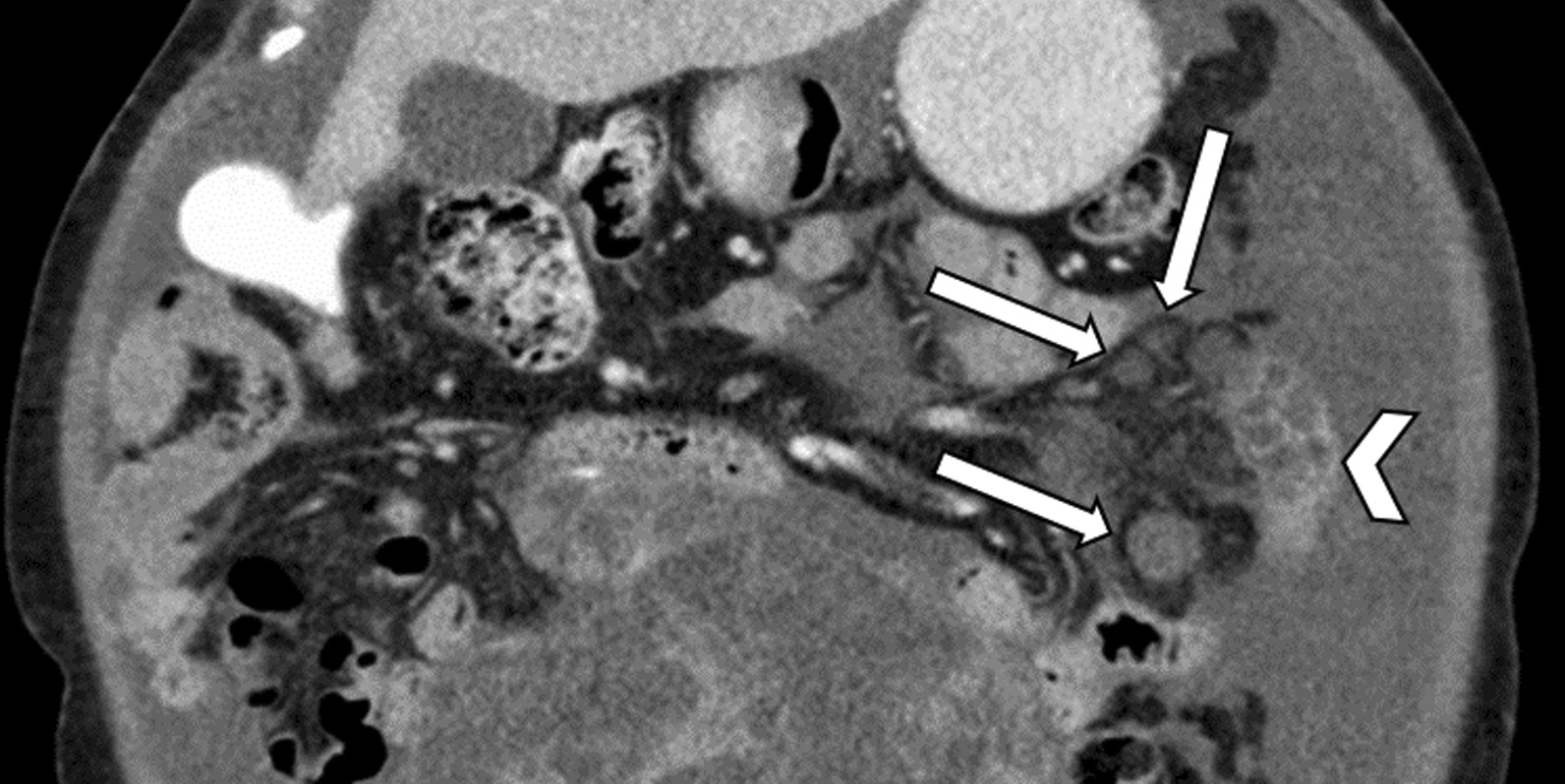
This portion of an abdominopelvic CT taken prior to the preexploratory laparotomy demonstrates hypodense spherical and ovoid locules (arrows) within the mesentery and adjacent to the small bowel (arrowhead)

A 61-year-old Caucasian woman presented with 5-year history of abdominal pain and distension, fatigue, and 1-year history of abnormal uterine bleeding. Initial workup revealed a carcinoembryonic antigen value of 8.1 ng/mL (normal < 3.9 ng/mL), cancer antigen-125 value of 134 U/mL (normal < 38.1 ng/mL), and a large left adnexal mass on ultrasound. Preoperative CT of the patient’s abdomen and pelvis without oral contrast initially revealed hypodense, ovoid lesions in the left lower quadrant mesentery adjacent to a short segment of small bowel (Fig. 1).

**Fig. 2.**
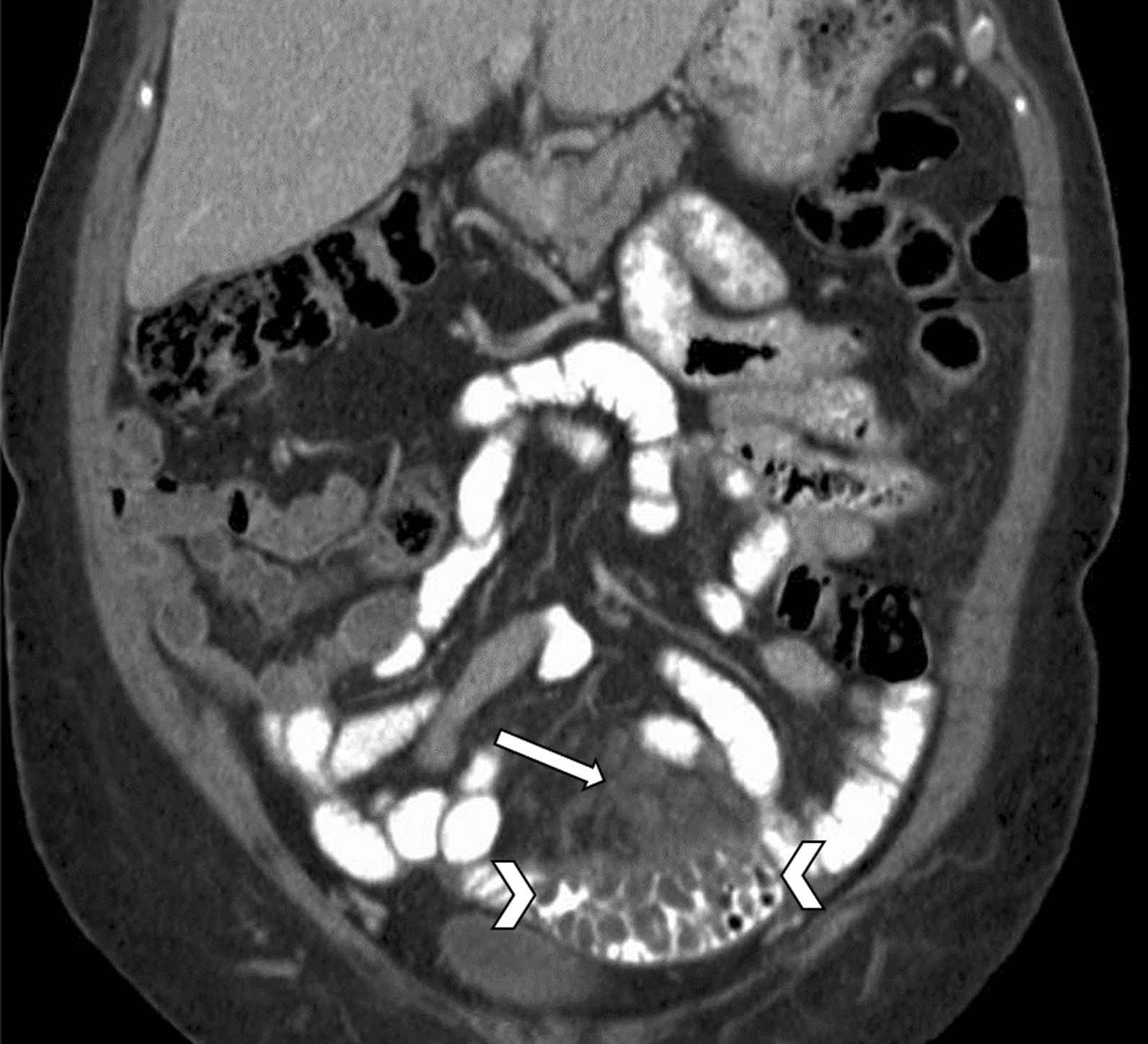
Abdominopelvic CT demonstrating an 8-cm segment of small bowel with low-density mural wall thickening (arrowheads) and multiple grape-like ovoid hypodense mesenteric fluid-containing structures (arrow)

The patient underwent an exploratory laparotomy which confirmed a diagnosis of mucinous ovarian carcinoma. One-month postoperative surveillance CT of the abdomen and pelvis with oral contrast demonstrated marked decrease in ascites and a 7–8-cm segment of small bowel in the left lower quadrant with low-density mural wall and bowel fold thickening. Additionally, there were hypodense ovoid lesions in the adjacent mesentery, which are the same lesions seen on the preoperative CT (Fig. 2). These findings were thought to represent persistent presence of peritoneal and serosal tumor implants.

The patient subsequently began chemotherapy with folinic acid, fluorouracil, and oxaliplatin (FOLFOX) and completed two treatment cycles. Trastuzumab was then added, and the patient underwent an additional three cycles of FOLFOX–trastuzumab. A repeat abdominopelvic CT 4 months after the exploratory laparotomy showed resolution of ascites, but redemonstrated unchanged findings in the short segment of small bowel and adjacent mesentery in the left lower quadrant. These findings were again thought to represent persistent peritoneal and serosal tumor implants despite chemotherapy.

**Fig. 3.**
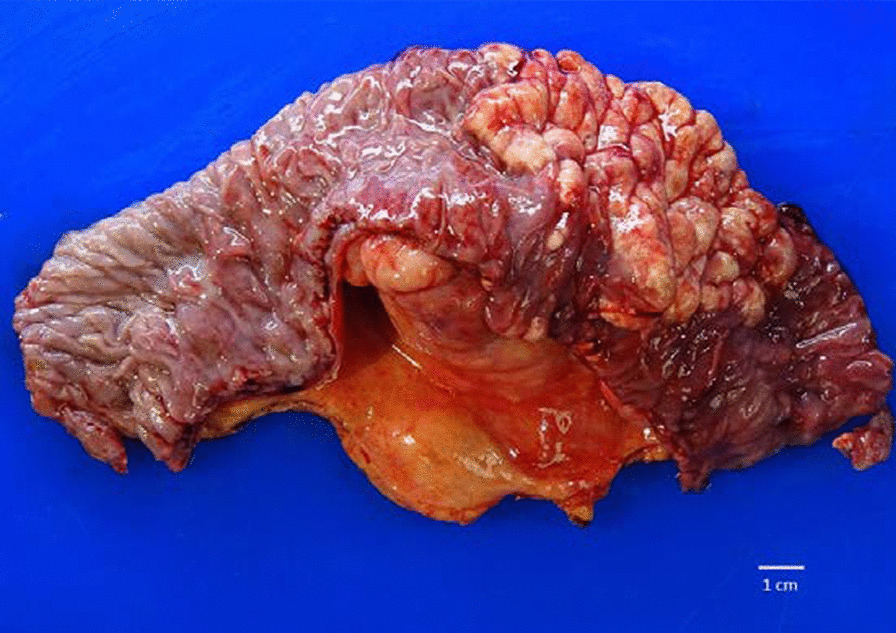
Lymphatic malformation. The gross photograph demonstrates a portion of jejunum with an 8-cm tan–white, irregular, nodular, mucosal lesion

**Fig. 4.**
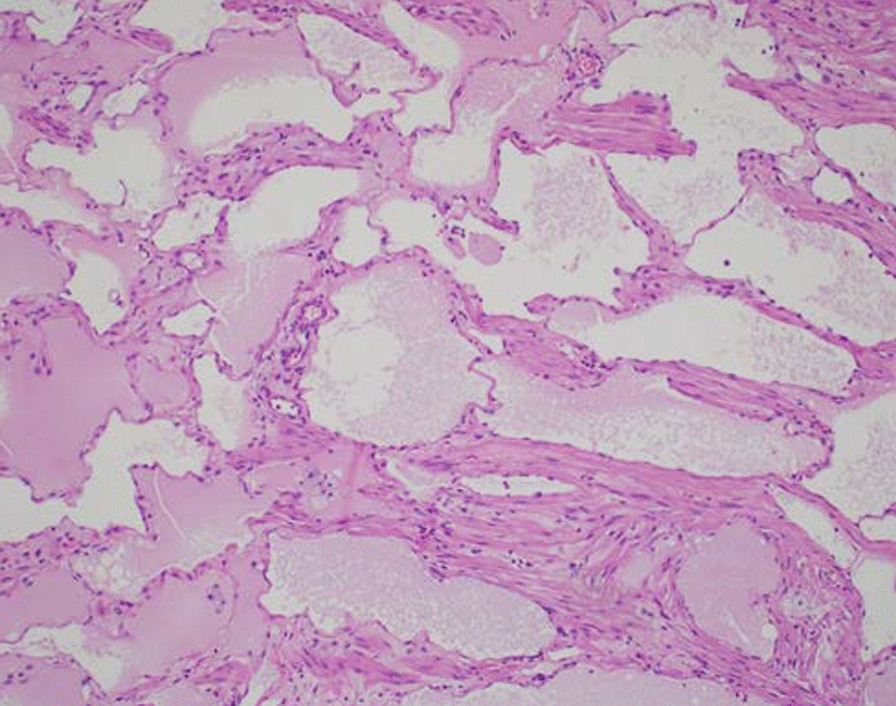
Lymphatic malformation at high power (H&E). The image demonstrates dilated lymphatic channels of varying sizes, lined by a single layer of endothelial cells. Disorganized smooth muscle is focally present

Shortly after the repeat CT, the patient underwent extensive tumor debulking. Intraoperatively, an 8-cm segment of jejunum with rubbery-like texture and adjacent mesentery was excised (Fig. 3). Histologic examination revealed a lymphatic malformation with variously sized lymphatic channels lined by a single, continuous layer of endothelia (Fig. 4). No residual intraperitoneal tumor was identified. The patient was subsequently discharged with a plan to complete FOLFOX–trastuzumab chemotherapy for her metastatic ovarian carcinoma.

## Discussion

Less than 1% of lymphatic malformations occur in the small bowel [4]. While no consensus exists for their development, they likely form when isolated lymphatic channels fail to connect appropriately to more prominent main channels, but evidence suggests that they can also be acquired from traumatic, inflammatory, or iatrogenic processes [5]. In adults, case reports indicate a variable presentation ranging from asymptomatic incidental findings [6] to acute [7] or chronic [8] abdominal pain, gastrointestinal bleeding [9], intussusception [4], and volvulus [10].

When imaged on CT, lymphatic malformations are typically described as cystic mesenteric structures filled with hypodense fluid that compress immediately adjacent bowel [8]. Contrast enhancement may reveal the presence of septae [2], but the cystic portion of the malformation is either filled with nonenhancing fluid, or rarely, with hemorrhage or calcifications [12].

Our patient’s lymphatic malformation had several features that mimicked peritoneal and serosal mucinous tumor implants. Tumor implants typically show thicker walls and septa, “scalloping” of visceral surfaces due to compressive forces from mucin produced by the peritoneal implants, and heterogeneous internal densities [13]. The ovoid mesenteric fluid-containing structures in our patient were part of the lymphatic malformation; however, in the setting of mucinous ovarian tumor, they mimicked peritoneal carcinomatosis with trapped malignant ascites. Additionally, lymphatic fluid within the jejunal wall likely caused the hypodense intramural thickening that mimicked serosal tumor implants.

Given the rarity of lymphatic malformations of the bowel and mesentery, there are few radiology studies devoted to diagnosis and characterization; however, contrast-enhanced magnetic resonance imaging may be a potentially useful test in distinguishing lymphatic malformations from mucinous tumor implants [14]. Lymphatic malformations typically have high signal intensity on T2-weighted imaging and lower internal T1 signal [2]. Mucinous tumors, on the other hand, typically have internally heterogeneous T1 and T2 signal intensities, although tumors containing a high concentration of mucin could display higher T1 and lower T2 signal intensities [15].

Gastrointestinal lymphatic malformations are typically treated with surgical removal considering their potential for mass effects, although sclerotherapy [16] and lymphovenous anastomosis [17] have been successfully reported. Histologic examination of the removed malformations typically show dilated lymphatic channels with flat, continuous endothelia [3]. They can be classified as macrocystic, microcystic, or mixed [19] depending on their microscopic size, although this classification is difficult to interpret radiologically.

## Conclusion

Lymphatic malformations of the small bowel are exceptionally rare anomalous developments of the lymphatic system. In the setting of mucinous tumors, lymphatic malformations with their variable presentation and nonspecific imaging appearance can present a diagnostic challenge as they mimic tumor deposits. Increased awareness of this entity may help improve patient care.

## Data Availability

Not applicable.

## Abbreviations

CT: Computed tomography
FOLFOX: Folinic acid, fluorouracil, oxaliplatin
